# From iPSCs to myotubes: Identifying potential biomarkers for human FSHD by single‐cell transcriptomics

**DOI:** 10.1002/ctm2.70423

**Published:** 2025-07-29

**Authors:** Wenwen Liu, Hao Chen, Jiao Jiao, Qinxin Zhang, Dong Liang, Haiqin Huo, Xiuqing Ji, Mingtao Huang, Xiaojing Hou, Yan Cao, Sihui Wu, Yan Wang, Jun Zhang, Zhengfeng Xu, Ping Hu

**Affiliations:** ^1^ Department of Prenatal Diagnosis Women's Hospital of Nanjing Medical University Nanjing Women and Children's Healthcare Hospital Nanjing China; ^2^ Nanjing Women and Children's Healthcare Institute Women's Hospital of Nanjing Medical University, Nanjing Women and Children's Healthcare Hospital Nanjing China; ^3^ State Key Laboratory of Reproductive Medicine and Offspring Health Nanjing Medical University Nanjing China; ^4^ Jiangsu Provincial Key Laboratory of Biological Therapy for Organ Failure Nanjing Medical University Nanjing China

1

Dear Editor,

Facioscapulohumeral muscular dystrophy (FSHD), an autosomal dominant neuromuscular disorder, exists as two molecular subtypes: FSHD1 (95% of cases), defined by pathogenic contraction of the 4q35‐located D4Z4 macrosatellite repeat,^1^ and FSHD2 (5%), caused by loss‐of‐function mutations in chromatin‐modifying suppressors (e.g., *SMCHD1*, *DNMT3B* and *LRIF1*).^2^ Both subtypes converge on D4Z4 hypomethylation‐mediated epigenetic derepression of *DUX4* gene, whose aberrant expression drives myocyte apoptosis and inflammatory cascades in terminally differentiated muscle.^3^ The scarcity of DUX4 expression and limited accessibility to viable muscle biopsies underscore the utility of patient‐derived induced pluripotent stem cells (iPSCs), which maintain donor‐specific genetic/epigenetic profiles, as physiologically relevant in vitro models for FSHD pathomechanistic studies. Here, we employed iPSC‐derived myotubes to investigate the pathogenesis of FSHD via single‐cell transcriptomic analysis, with the aim of identifying novel biomarkers.

We established in vitro FSHD models by differentiating iPSCs from healthy controls (HCs) and FSHD1 patients into myogenic progenitors and myotubes via Wnt activation and BMP inhibition (Figure 1A), utilising an established differentiation protocol.^4^ Peripheral blood mononuclear cell‐derived iPSCs exhibited pluripotency markers (OCT4/NANOG; Figure 1B) and underwent sequential differentiation into PAX7^+^/MYOD1^+^ progenitors (Figure 1C) and multinucleated myotubes expressing skeletal muscle markers (MF20/α‐actinin; Figure 1D). While comparative analysis revealed comparable expression of myogenic surface markers (CD82/CD56) and myotube maturation (MF20^+^) between FSHD1 and HC groups (Figure 1E‒G), FSHD1 myotubes exhibited specific upregulation of DUX4 and its transcriptional targets^5^ (*MBD3L2*, *ZSCAN4* and *TRIM43*; Figure 1H,I), recapitulating disease‐specific transcriptional dysregulation within a conserved differentiation framework.

**FIGURE 1:**
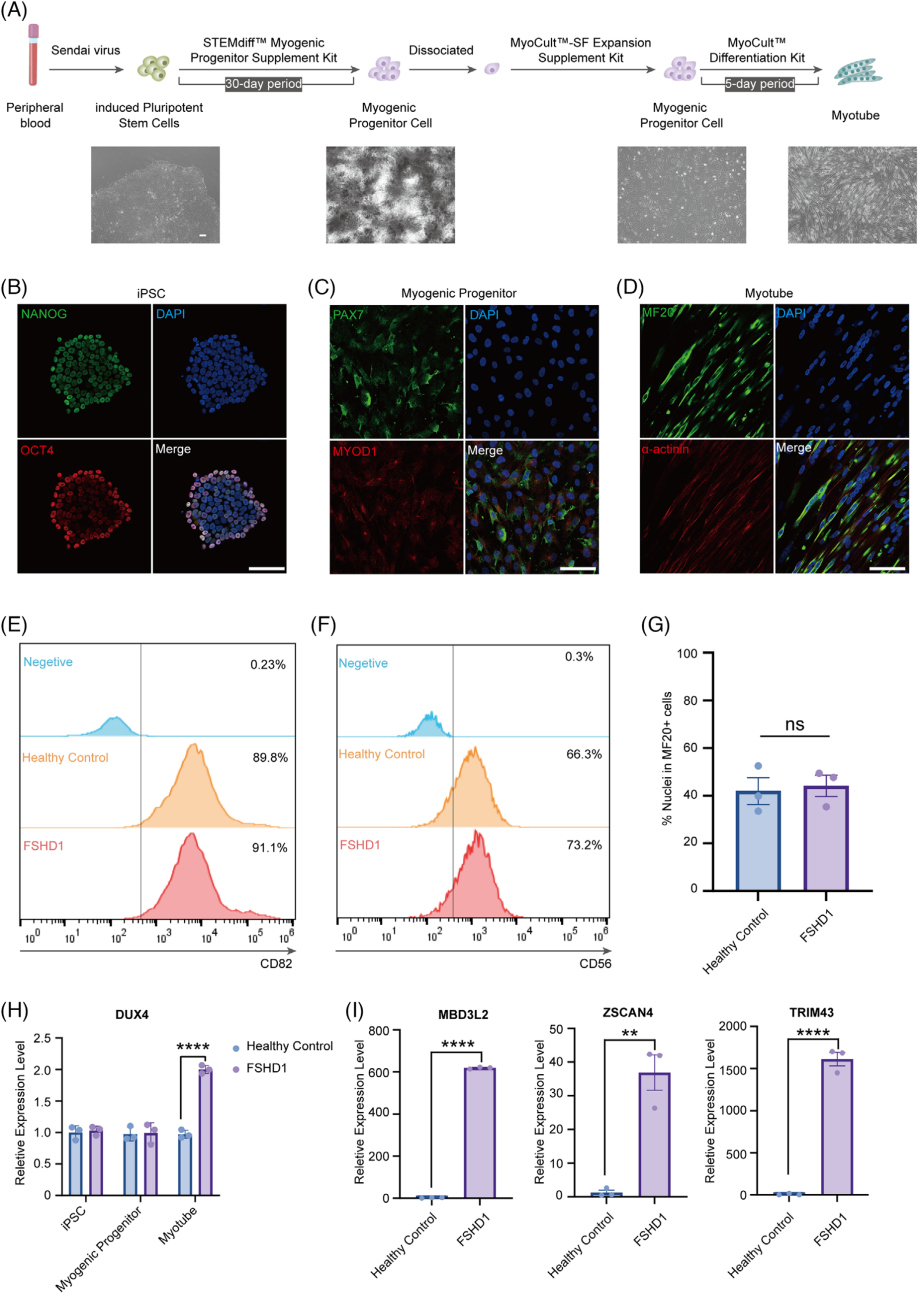
Induced pluripotent stem cells (iPSCs) were induced to differentiate into myogenic progenitor cells and myotubes in vitro. (A) Flow chart of myogenic progenitor cells and myotubes induced from iPSCs. In our culture system, we initially reprogrammed peripheral blood mononuclear cells (PBMCs) by using a Sendai virus vector to generate iPSCs. Subsequently, iPSCs were differentiated into myogenic progenitor cells (hPSC‐MPs) by using the STEMdiff Myogenic Progenitor Supplement Kit (Stemcell, #100‐0151) over a 30‐day period. On the 30th day, the cultured cells were dissociated into a single‐cell suspension and expanded using the MyoCult‐SF Expansion Supplement Kit (Stemcell, #05980), leading to the formation of tubular structures. The expanded myogenic progenitor cells were then differentiated using the MyoCult Differentiation Kit (Stemcell, #05965). By the fifth day of differentiation, the cells were arranged in bundled structures and exhibited typical morphology of a myotube. (B) Immunofluorescence staining results of iPSC surface markers OCT4 and NANOG. Scale bars = 100 µm. DAPI stands for staining of the nucleus. (C) Immunofluorescence staining results of MYOD1 and PAX7 markers on myogenic progenitor cell. Scale bars = 100 µm. (D) Immunofluorescence staining results of myotubes surface marker α‐actinin and MF20. Scale bars = 100 µm. (E) Flow cytometry assessed the proportion of CD82‐positive cells in myogenic progenitor cells from healthy controls and facioscapulohumeral muscular dystrophy 1 (FSHD1) patients, using blue for the negative reference, orange for healthy controls and red for FSHD1. The right value indicates the proportion of positive cells in each sample. (F) Flow cytometry evaluated the proportion of CD56‐positive cells in myogenic progenitor cells from healthy controls and FSHD1 patients, with blue as the negative reference, orange for healthy controls and red for FSHD1. The right value reflects the proportion of positive cells in each sample. (G) Percentage of MF20‐positive cells in healthy controls and FSHD1 differentiated myotubes. ns: no significance. (H) The relative expression level of *DUX4* gene in iPSC, myogenic progenitor cells and myotubes of healthy control group and FSHD1 group was detected by qPCR. ^****^ *p* < .0001. (I) The relative expression levels of DUX4 target genes *MBD3L2*, *ZSCAN4* and *TRIM43* in healthy controls and FSHD1 myotubes were detected by qPCR. ^**^ *p* < .01; ^****^ *p* < .0001.

Single‐cell RNA sequencing (scRNA‐seq) was performed to resolve transcriptional dynamics during iPSC‐derived myogenesis, analysing 132 482 cells (HCs: 65 266; FSHD1: 67 216) across differentiation stages (progenitors, myotubes day 3/day 5) obtained from sex‐matched FSHD1 donors with distinct clinical‐genetic profiles: a 26‐year‐old male carrying contracted D4Z4 repeats (4 units at 4q35) and a female patient diagnosed at age 6 with severe allelic contraction (2 repeat units) (Figure S1). Based on the gene signatures,^6^, ^7^ we identified 13 major cell types, including muscle satellite cells‐1/2 (*CDCP1*), PAX3^+^ myogenic progenitors‐1/2 (*PAX3*), proliferating PAX3^+^ myogenic progenitors (*PAX3*/*MKI67*), PAX7^+^ myogenic progenitors (*PAX7*), proliferating PAX7^+^ myogenic progenitors (*PAX7*/*MKI67*), myofibroblasts, myoblasts (*MYOD1*/*MYOG*), neuronal contaminants (*ELAVL4*) and three myotube clusters (*TTN*) (Figure 2A,B). Myotube clusters exhibited stage‐specific differentiation signatures: MYH2^+^ MYH7^+^ myotubes expressed adult skeletal muscle myosin isoforms MYH2/MYH7, while MYMK^high^ MYMX^high^ myotubes and MYMK^low^ MYMX^low^ myotubes reflected sequential myoblast fusion kinetics mediated by transient MYMK/MYMX dynamics,^8^ consistent with their roles in initiating semi‐fusion and terminal multinucleation, respectively. scRNA‐seq delineated stage‐specific transitions during in vitro iPSC‐myotube differentiation, revealing progressive satellite cell depletion, PAX3^+^ progenitor expansion, and myotube diversification across progenitor, intermediate (myotube D3) and terminal (myotube D5) maturation phases (Figure S2A‒C). Gene Ontology (GO) enrichment analysis revealed distinct biological processes across subpopulations: muscle satellite cells and PAX3^+^ myogenic progenitors were enriched for cell adhesion and extracellular matrix organisation; proliferating myogenic progenitors for cell cycle regulation, DNA replication and mitosis; and myotubes for skeletal muscle differentiation processes including sarcomere organisation and muscle contraction (Figure 2C). Notably, cluster 11 showed significant enrichment for nervous system development—consistent with established literature demonstrating neural progenitor emergence in prolonged myogenic differentiation cultures^4^—reflecting Wnt/BMP‐mediated neuronal lineage specification in hiPSC‐derived innervated muscle systems.^9^

**FIGURE 2:**
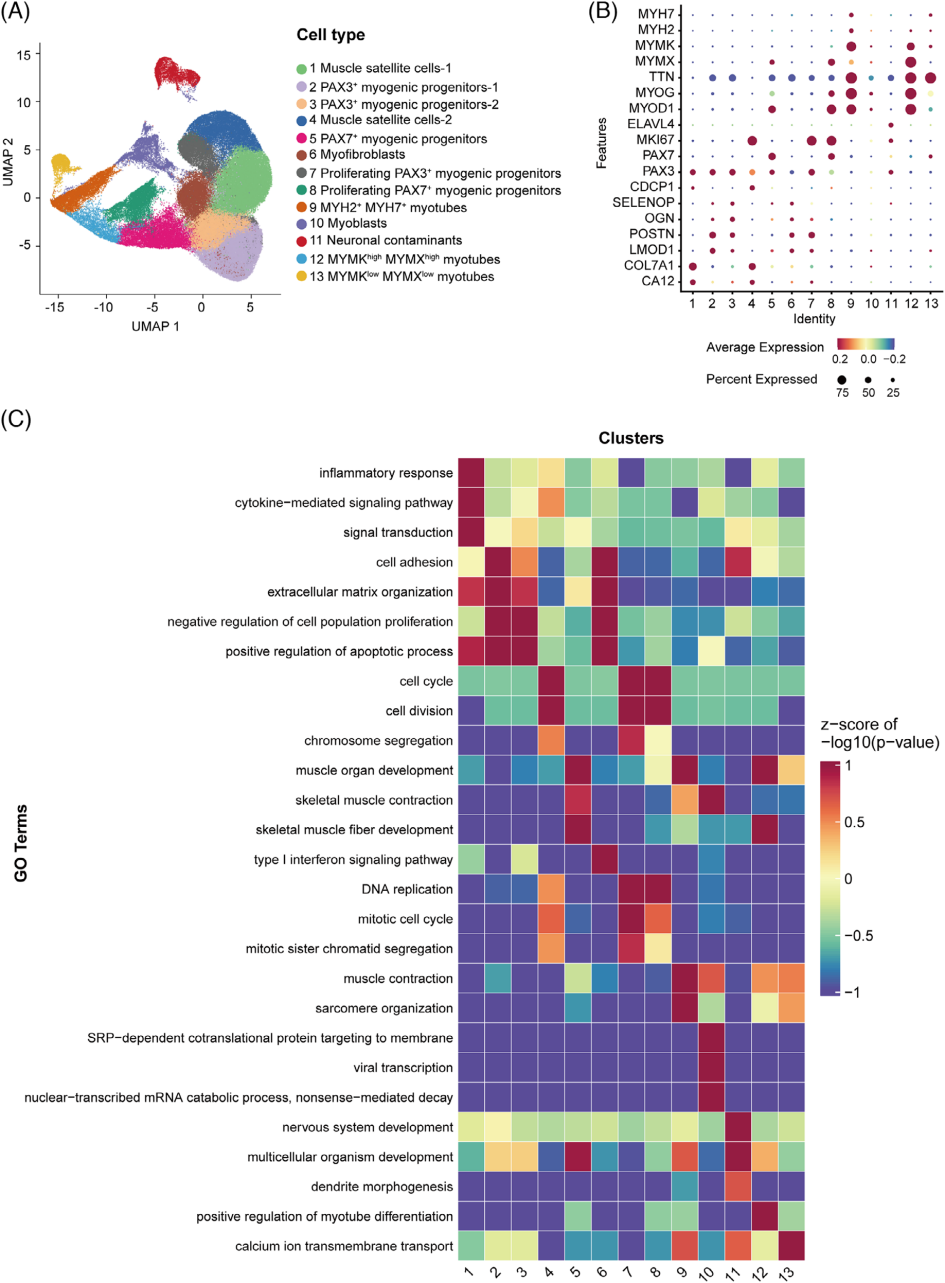
Identifying cell types in induced pluripotent stem cells (iPSC)‐derived myogenic progenitor cells and myotubes using single‐cell transcriptomic profiling. (A) iPSC‐induced myogenic progenitor cells and myotube cultures were segregated into distinct clusters in the UMAP plot. (B) Dot plots of marker gene expression levels in 13 cell clusters from myogenic progenitor cells and myotube cultures, with blue to red colours indicating low to high expression. Dot size represents the proportion of cells expressing each gene. (C) Heatmap visualisation of non‐redundant Gene Ontology (GO) biological process enrichments for top marker genes across cell subpopulations. Top 3 significant terms per cluster (*p*‐value ranked) are displayed.

RNA velocity analysis via scVelo mapped differentiation trajectories of iPSC‐derived myogenesis (Figure S3A). Vector field modelling identified lineage progression from satellite cells to PAX3^+^ progenitors (pathway ①), followed by PAX3^+^‐to‐PAX7^+^ progenitor expansion (pathway ②) and satellite cells to proliferating PAX3^+^ progenitors (pathway ③). Transcriptional dynamics transitioned from myoblast‐to‐myotube differentiation at myotube D3 (pathway ④) to terminal commitment at myotube D5 (pathway ⑤), with attenuated progenitor‐driven dynamics and residual PAX7^+^ autodifferentiation (Figure 3A). The latent time, which represents pseudo‐time within cells, approximates the transcriptional dynamics during cell differentiation (Figure S3B), and our analysis identified mitosis‐related drivers (e.g., *FGFR4*, *CENPF* and *AURKA*) and skeletal muscle function regulators (e.g., *ARPP21*, *TNNT1* and *BIN1*) governing differentiation‐stage transitions (Figure S3C).

**FIGURE 3:**
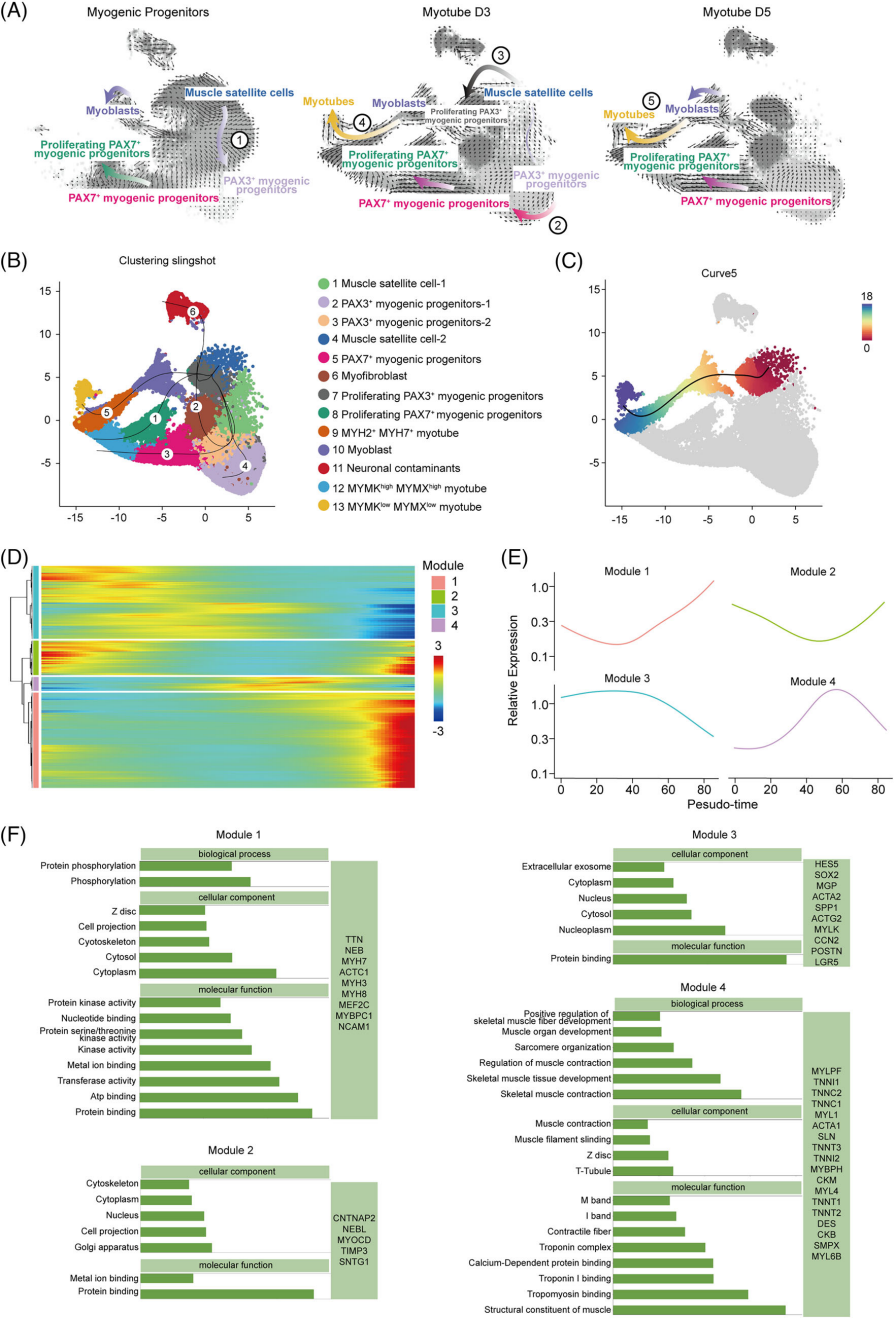
Delineate the lineage differentiation of myogenic progenitor cells into myotubes, explain cell fate and transcriptional activity through scVelo analysis and Slingshot's inferred differentiation lineage of muscle satellite cells into mature myotubes. (A) Simplified five streamlines of differentiation process according to gene velocity of different stages of differentiation of myogenic progenitor cells and myotubes. (B) Slingshot analysis inferred six differentiation paths from muscle satellite cells to MYMK^low^MYMX^low^ myotubes. (C) Pseudo‐temporal inference results of differentiation trajectory curve 5. Dots in the figure represent cells, solid black lines represent cell differentiation trajectories, colours ranging from red to blue indicate pseudo‐time from early to late differentiation, and grey represents cells that do not belong to this lineage. (D) The heatmap of four modules with different expression trends were identified by clustering analysis of cell expression profiles. (E) Four different trend plots based on cell expression profiles. (F) GO cluster analysis of differential genes of four different expression trend modules identified by cell expression profile cluster analysis, and display muscle‐related functions and genes.

Slingshot trajectory analysis revealed six differentiation patterns, with curve 5 exhibiting UMAP‐defined start/end points most aligned with myogenic progression (Figure 3B,C). Monocle‐based pseudo‐temporal ordering of curve 5 clusters—including satellite cells‐2 (cluster 4), PAX3^+^ progenitors (cluster 7), myoblasts (cluster 10) and distinct myotube subtypes (clusters 9/13)—identified a branching point defining three states: early (clusters 4/7), transitional (myoblasts spanning states 2–3) and terminal (clusters 9/13) (Figure S4A‒E), consistent with scVelo dynamics. Pseudo‐temporal analysis of 9126 Slingshot‐inferred curve 5 cells identified four co‐expression modules: module 1 (late‐upregulated) encoded Z‐disc/cytoskeletal components (e.g., *TTN*, *NEB*, *MYH7* and *MYH3*); module 2 (biphasic) involved contraction‐related projections (e.g., *CNTNAP2*, *NEBL*, *MYOCD*, *TIMP3* and *SNTG1*); module 3 (early‐downregulated) contained pluripotency factors (*SOX2*); module 4 (terminal‐downregulated) regulated myotubes maturation (e.g., *MYLPF*, *TNNI1*, *TNNC2*, *TNNC1* and *MYL1*) (Figures 3D‒F and S4F,G).

To assess our iPSC‐derived myogenic differentiation platform—developed from non‐muscle tissue sources—for modelling FSHD pathology and establishing potential biomarkers, we conducted comparative transcriptomic analyses between FSHD patients and HCs by intersecting differentially expressed genes with established FSHD‐associated gene signatures. Interrogation of the 67‐gene DUX4 biomarker panel (DUX4‐67)^10^ revealed a minor DUX4‐affected subpopulation (six cells) exclusively within myotube D3 (FF2 sample) of the female FSHD, with no comparable signal detected in male FSHD or HCs (Figure 4A,B). Subsequent analysis of FSHD1‐specific (27 genes)^10^ and DiffExpr‐CoreSet113 signatures^10^ identified 36 significantly upregulated genes in FSHD, comprising two from the former and 34 from the latter. The FF2 female FSHD myotube sample exhibited pronounced DUX4 biomarker expression, featuring 32 CoreSet genes that robustly indicate DUX4 activity (Figure 4C). Collectively, these findings demonstrate that this iPSC‐based myogenic platform recapitulates hallmark molecular features of FSHD, establishing its utility for mechanistic investigation of disease pathogenesis.

**FIGURE 4:**
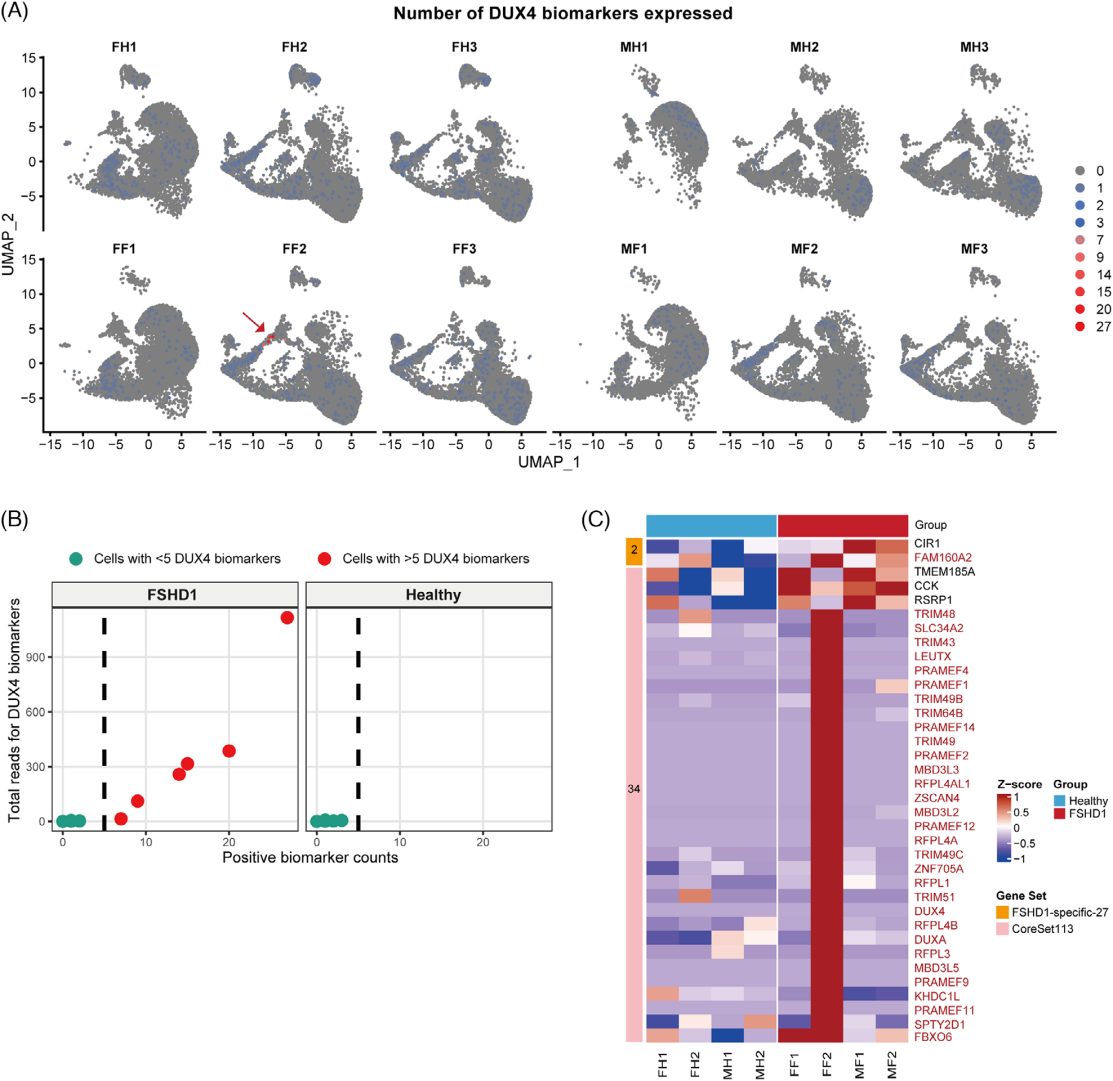
Single‐cell transcriptomic interrogation of DUX4 target signatures in facioscapulohumeral muscular dystrophy (FSHD). (A) Cellular expression frequency of DUX4‐67 signature genes across 12 samples. Arrow denotes DUX4‐affected subpopulation. (B) Relationship between expressed DUX4 biomarkers per cell and aggregate transcript counts in FSHD specimens. Dashed line indicates threshold for DUX4‐affected cells (≥5 biomarkers/cell). (C) Heatmap of FSHD1‐Specific27 (orange box, *n* = high‐expression genes) and DiffExpr‐CoreSet113 (pink box, *n* = high‐expression genes) signatures in myogenic progenitors and myotube D3. Values represent row‐normalised mean transcript abundance per gene across sample‐specific populations. Red‐labelled genes denote 32 CoreSet markers robustly indicating DUX4 activity.

This study establishes an iPSC‐derived myotube model that recapitulates FSHD pathogenesis at single‐cell resolution, resolving 13 transcriptionally distinct clusters and molecular signatures. Pseudo‐temporal analysis validated conserved myogenic trajectories across samples. Although cohort size was limited, analysis of representative FSHD cases revealed robust DUX4 target gene activation through concordance with established DUX4 biomarkers. This platform provides a foundational tool for dissecting FSHD pathogenesis and advancing precision therapeutic strategies.

## AUTHOR CONTRIBUTIONS

Ping Hu, Zhengfeng Xu, Jun Zhang, Yan Wang, Wenwen Liu, Hao Chen and Jiao Jiao conceived the ideas and designed the project. Jiao Jiao, Wenwen Liu, Hao Chen, Qinxin Zhang, Yan Wang, Dong Liang, Haiqin Huo, Xiuqing Ji, Xiaojing Hou, Yan Cao and Sihui Wu performed most of the experiments. Wenwen Liu and Mingtao Huang performed bioinformatics analysis. Ping Hu and Zhengfeng Xu provided funds. Wenwen Liu, Jiao Jiao and Ping Hu wrote the manuscript. All the authors have read and approved the final manuscript.

## CONFLICT OF INTEREST STATEMENT

The authors declare they have no conflicts of interest.

## FUNDING INFORMATION

This work was supported by National Key R&D Program of China (no. 2022YFC2703400 to PH and ZX and no. 2021YFC1005301 to PH), the National Natural Science Foundation of China (no. 82371862 to PH) and Nanjing Health Science and Technology Development Special Fund Project (no. YKK24148 to WL).

## ETHICS STATEMENT

This study was ethically approved by Women's Hospital of Nanjing Medical University (2021KY‐123). All the participants provided informed consent during sample collection, with de‐identified data.

## Supporting information



Supporting Information

## Data Availability

The raw sequence data of scRNA‐seq reported in this paper have been deposited in the Genome Sequence Archive in National Genomics Data Center, China National Center for Bioinformation/Beijing Institute of Genomics, Chinese Academy of Sciences (GSA‐Human: HRA008120) that are publicly accessible at https://ngdc.cncb.ac.cn/gsa‐human. These data are available on request from the corresponding author upon reasonable request.
