# Supplementary material for: From iPSCs to myotubes: Identifying potential biomarkers for human FSHD by single‐cell transcriptomics
Source: Clin Transl Med. 2025 Jul 29;15(8):e70423. doi: 10.1002/ctm2.70423 (PMC12304728; doi:10.1002/ctm2.70423)
Supplement: Supplementary file 1 — Supporting Information [file CTM2-15-e70423-s001.docx]

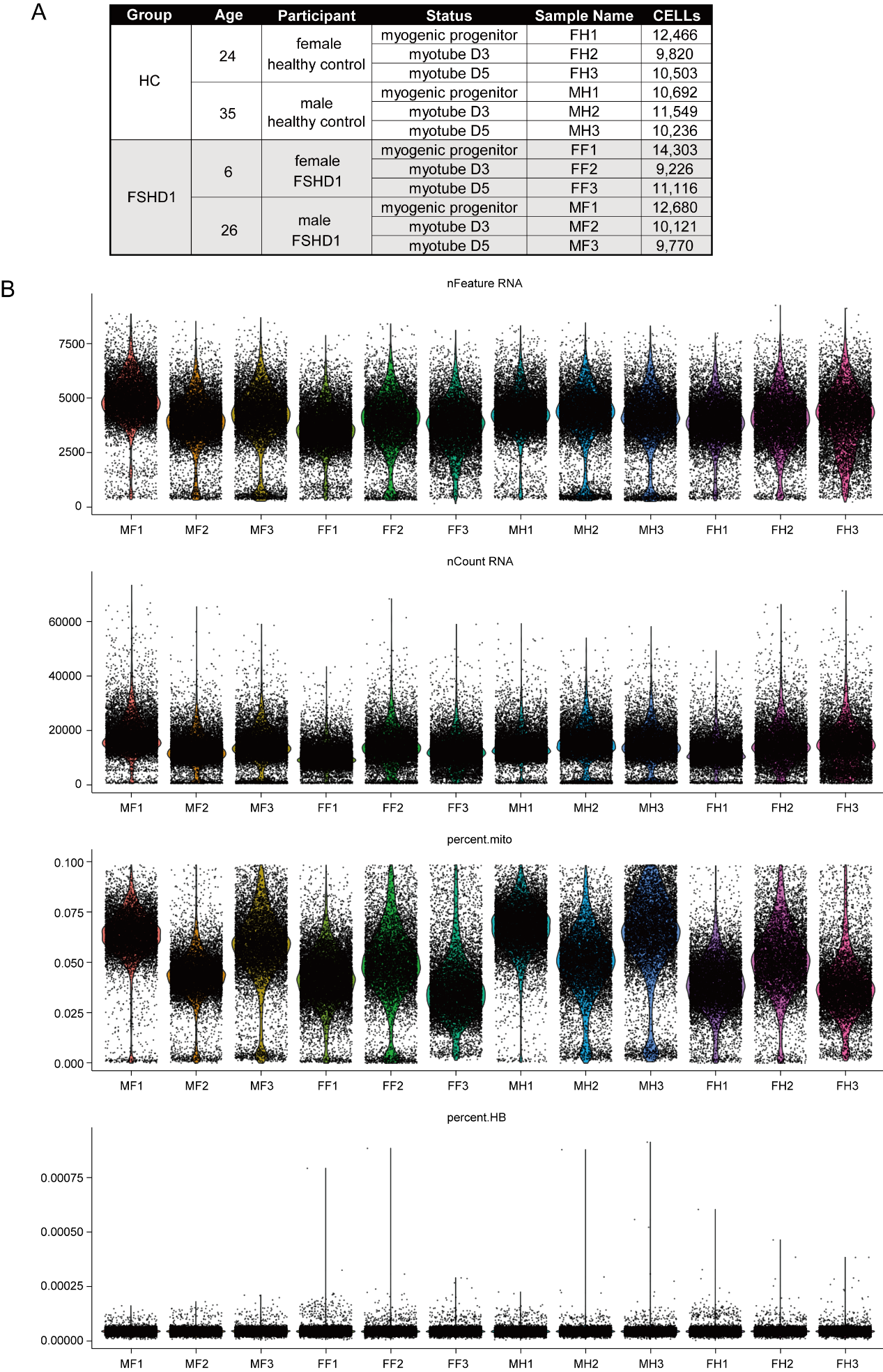


Supplementary Figure 1

1. The number of cells detected by scRNA-seq in myogenic progenitor cells and myotube cultures from healthy controls and FSHD1 patients.
2. Violin distribution map of each index content in each cell after quality control. Cells satisfied criteria that included nFeature＞200, nCount＞1000, percentage of mitochondria＞10%, and red blood cell gene＞5%.


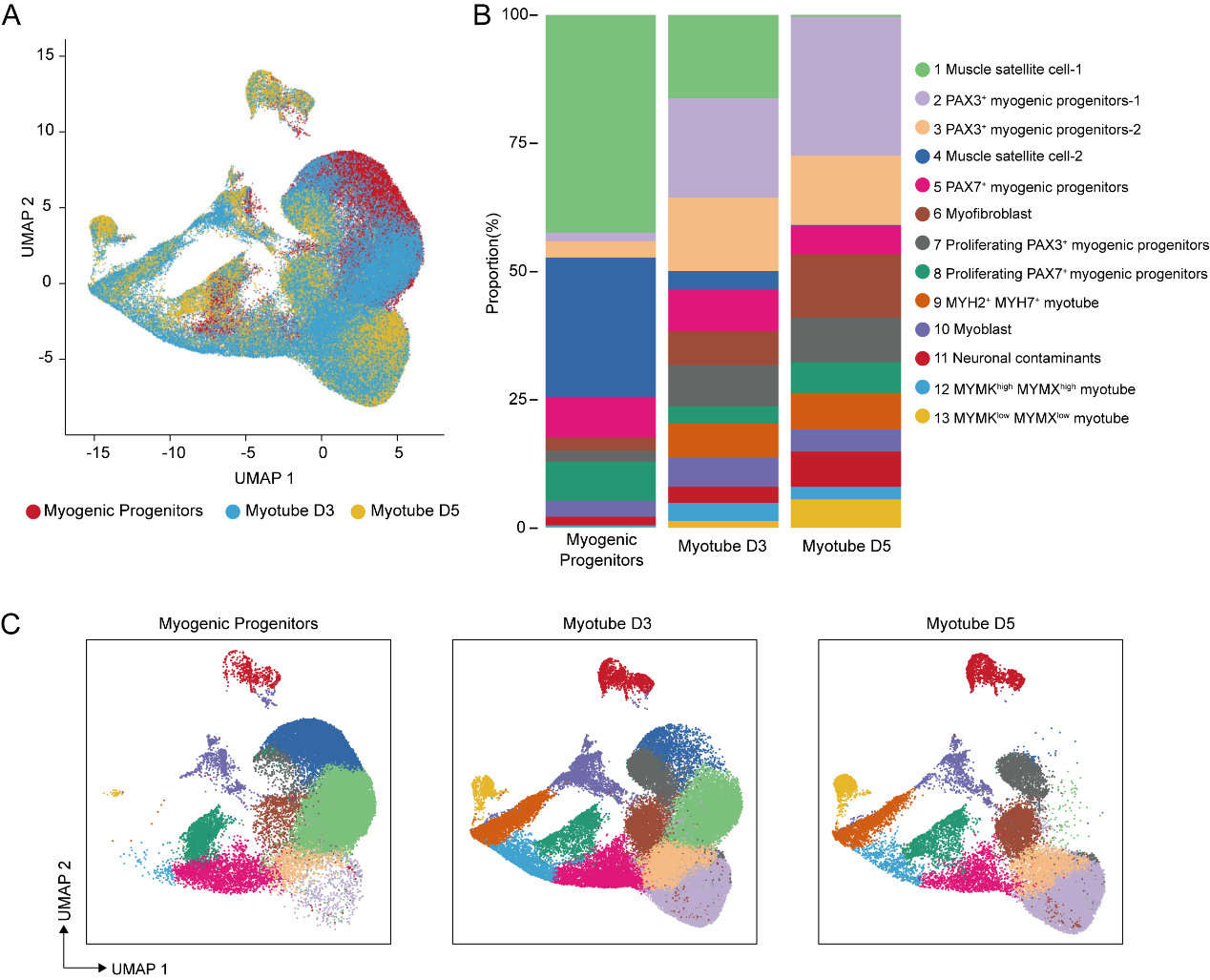


Supplementary Figure 2

1. UMAP representation of integrated all cell integrated from myogenic progenitor cells and myotube cultures.
2. The proportion of different cell clusters to the total number of cells in each group of myogenic progenitor cells and myotube cultures.
3. UMAP of myogenic progenitor cells and myotube cultures.


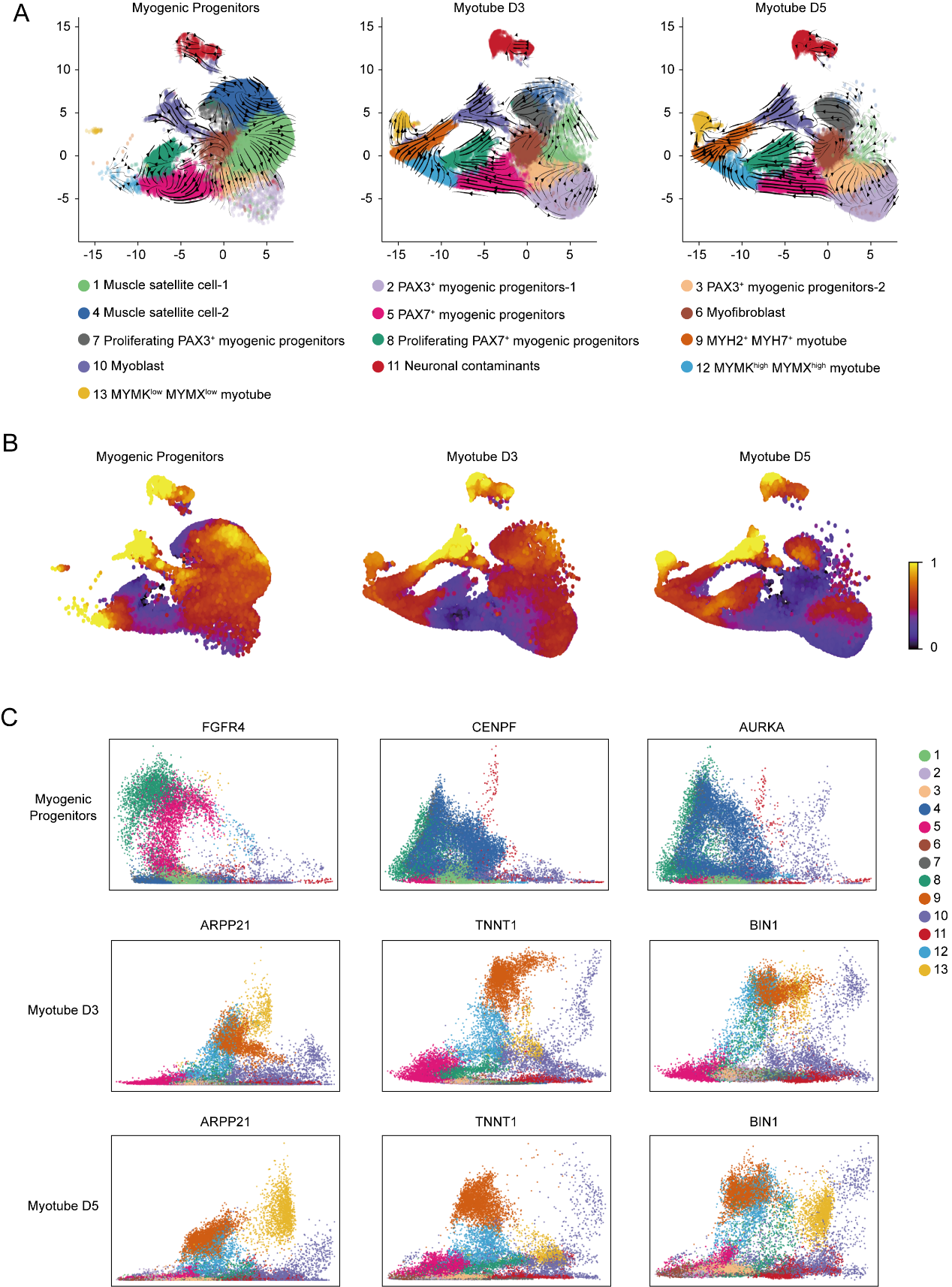


Supplementary Figure 3

1. Velocities derived from the dynamical model for myogenic progenitor cells into myotubes are visualized as streamlines in a UMAP-based embedding.
2. scVelo’s latent time of myogenic progenitor cells differentiating into myotubes. The differentiation latent time close to 0 indicates that the cell is approaching the initial state, and close to 1 indicates that the cell is approaching the terminal or specialized state.
3. Putative driver genes for myogenic progenitor cells (*FGFR4*, *CENPF*, and *AURKA*) to differentiate into myotubes (*ARPP21*, *TNNT1*, and *BIN1*).


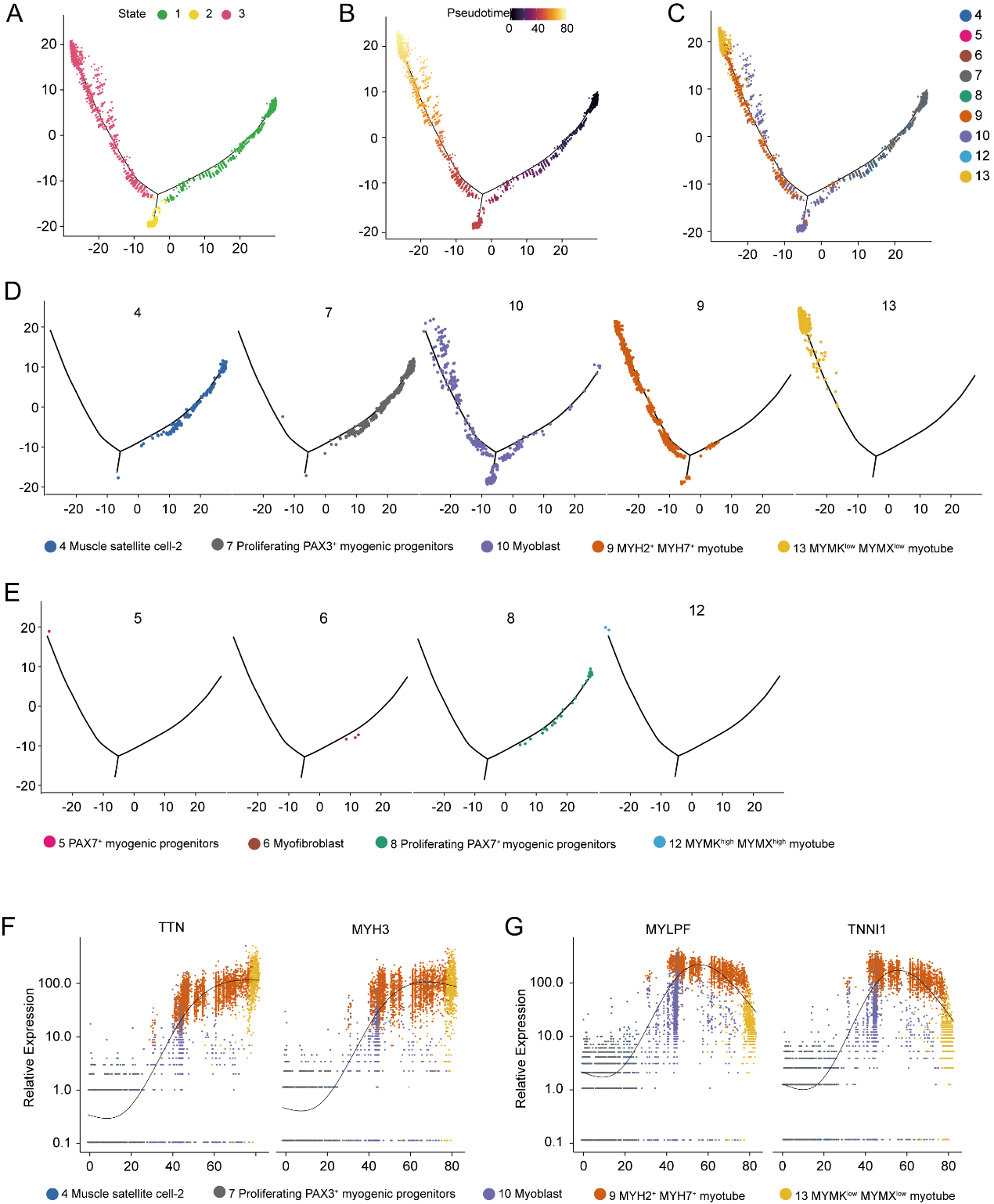
Supplementary Figure 4

1. Branch points of three cell states obtained by pseudo-temporal trajectory diagram of Cruve 5. Green represents differentiation stage 1, yellow represents differentiation stage 2 and red represents differentiation stage 3.
2. Pseudo-temporal time trajectory diagram of Cruve 5. The color is from dark to light, indicating that the time of differentiation is from early to late.
3. The distribution of each cell cluster in the pseudo-temporal trajectory diagram of Cruve 5.
4. UMAP of individual distribution of major cell clusters in Cruve5's pseudo-temporal trajectory.
5. UMAP of individual distribution of scattered cell clusters in Cruve5's pseudo-temporal trajectory diagram.
6. Expression trend diagram of the representative genes *TTN* and *MYH3* of Module 1.
7. Expression trend diagram of the representative genes *MYLPF* and *TNNI1* of Module 4.
